# Potential of digital chest radiography-based deep learning in screening and diagnosing pneumoconiosis: An observational study

**DOI:** 10.1097/MD.0000000000038478

**Published:** 2024-06-21

**Authors:** Yajuan Zhang, Bowen Zheng, Fengxia Zeng, Xiaoke Cheng, Tianqiong Wu, Yuli Peng, Yonliang Zhang, Yuanlin Xie, Wei Yi, Weiguo Chen, Jiefang Wu, Long Li

**Affiliations:** a Department of Radiology, Guangzhou Twelfth People’s Hospital, Guangzhou, China; b Department of Radiology, Nan fang Hospital, Southern Medical University, Guangzhou, China; c Department of Radiology, San shui District Institute for Disease Control and Prevention, Foshan Guangdong, China; d Department of Radiology, The Third People’s Hospital of Yunnan Province, Yunnan, China.

**Keywords:** deep learning, diagnosis, mass screening and classification, pneumoconiosis, radiography

## Abstract

The diagnosis of pneumoconiosis is complex and subjective, leading to inevitable variability in readings. This is especially true for inexperienced doctors. To improve accuracy, a computer-assisted diagnosis system is used for more effective pneumoconiosis diagnoses. Three models (Resnet50, Resnet101, and DenseNet) were used for pneumoconiosis classification based on 1250 chest X-ray images. Three experienced and highly qualified physicians read the collected digital radiography images and classified them from category 0 to category III in a double-blinded manner. The results of the 3 physicians in agreement were considered the relative gold standards. Subsequently, 3 models were used to train and test these images and their performance was evaluated using multi-class classification metrics. We used kappa values and accuracy to evaluate the consistency and reliability of the optimal model with clinical typing. The results showed that ResNet101 was the optimal model among the 3 convolutional neural networks. The AUC of ResNet101 was 1.0, 0.9, 0.89, and 0.94 for detecting pneumoconiosis categories 0, I, II, and III, respectively. The micro-average and macro-average mean AUC values were 0.93 and 0.94, respectively. The accuracy and Kappa values of ResNet101 were 0.72 and 0.7111 for quadruple classification and 0.98 and 0.955 for dichotomous classification, respectively, compared with the relative standard classification of the clinic. This study develops a deep learning based model for screening and staging of pneumoconiosis is using chest radiographs. The ResNet101 model performed relatively better in classifying pneumoconiosis than radiologists. The dichotomous classification displayed outstanding performance, thereby indicating the feasibility of deep learning techniques in pneumoconiosis screening.

## 1. Introduction

Pneumoconiosis is a chronic occupational lung disease caused by the inhalation of productive mineral dust. It is incurable and irreversible, and is the leading occupational disease in China.^[1]^ Chronic silicosis may develop or progress even after the cessation of occupational exposure; currently, there is no treatment other than a potential lung transplant.^[2–4]^ This necessitates diagnosing and classifying the stage of pneumoconiosis before its progress into an irreversible stage. To reduce complication rates and mortality, the International Labor Organization (ILO) recommends frequent pulmonary function tests and chest radiographs for people with occupational diseases.^[5]^ Researchers have developed a standardized system for classifying imaging abnormalities in pneumoconiosis according to the presence of the following pulmonary parenchymal and pleural abnormalities: small round turbidity, small irregular turbidity, massive turbidity, and other imaging features.^[6–8]^ X-ray imaging is the most common modality used in clinical settings worldwide.^[9]^

Currently, the clinical diagnosis of pneumoconiosis is principally based on the corrected interpretation of chest radiographs (X-ray images). Compared with standard radiographs, a radiologist assesses the concentration of small opacities on a chest X-ray image as category 0, I, II, or III.^[10]^ While classifying pneumoconiosis, we followed the ILO classification guidelines; nonetheless, we used standard radiographs collected and defined by the Chinese Center for Disease Control and Prevention.^[11,12]^ However, the diagnosis of pneumoconiosis remains challenging, is subjective, and varies among reviewers.^[13,14]^ To improve the diagnostic efficiency and accuracy among radiologists, researchers have developed computer-aided diagnosis (CAD) schemes for detecting pneumoconiosis using chest radiographs as a second opinion.

Several domestic and international scholars have studied the application of CAD technology in pneumoconiosis diagnosis.^[15,16]^ However, these traditional machine learning methods rely on the effectiveness of feature extraction and require “hand-crafted” feature recognition, which is technically time-consuming and labor-intensive, particularly for complex tasks, such as pneumoconiosis diagnosis and staging.^[17]^

Currently, metaheuristic algorithms are highly capable in solving different optimization problems due to their strong performance and speed. Some of these excellent algorithms are Grasshopper optimization algorithm (GOA).^[18]^ Particle swarm optimization (PSO)^[19]^ improved whale optimization salp swarm algorithm (IWOSSA),^[20]^ a hybrid of genetic algorithm and particle swarm optimization,^[21]^ enhanced Chimp-Harris Hawks optimization algorithm (ECH3OA).^[22]^ In addition in order to improve the metamorphic hair algorithm many learning techniques are also applied.

With the rapid development of deep learning technology, convolutional neural networks (CNNs) plays an important role in the diagnosis of different diseases, such as the diagnosis of cardiovascular disease,^[23]^ the detection of COVID-19,^[24]^ etc. Artificial Intelligence is not confined to medicine, but has been developed to all walks of life, and the algorithms are getting more and more mature and more advanced, with the rapid development of a class of deep learning algorithms represented by CNNs as well as the breakthroughs they have made in the task of computer vision,^[25]^ which has a lot of algorithms achieved very advanced state-of-the-art results, and the dataset has also achieved a very promising accuracy.

The deep learning technique has emerged as a novel and promising approach for solving challenging problems. Its advantage is that it implicitly learns complex imaging features or patterns without recognizing and extracting image features, which may involve tens of millions of features and analyze them to obtain high-level features.^[26,27]^ The deep learning network is an advanced technique that can study features and provide heat maps to visualize model decisions. This improves network reliability and accurately represents the input image, addressing the problem of the “black box” principle.^[28]^

**Figure 1.**
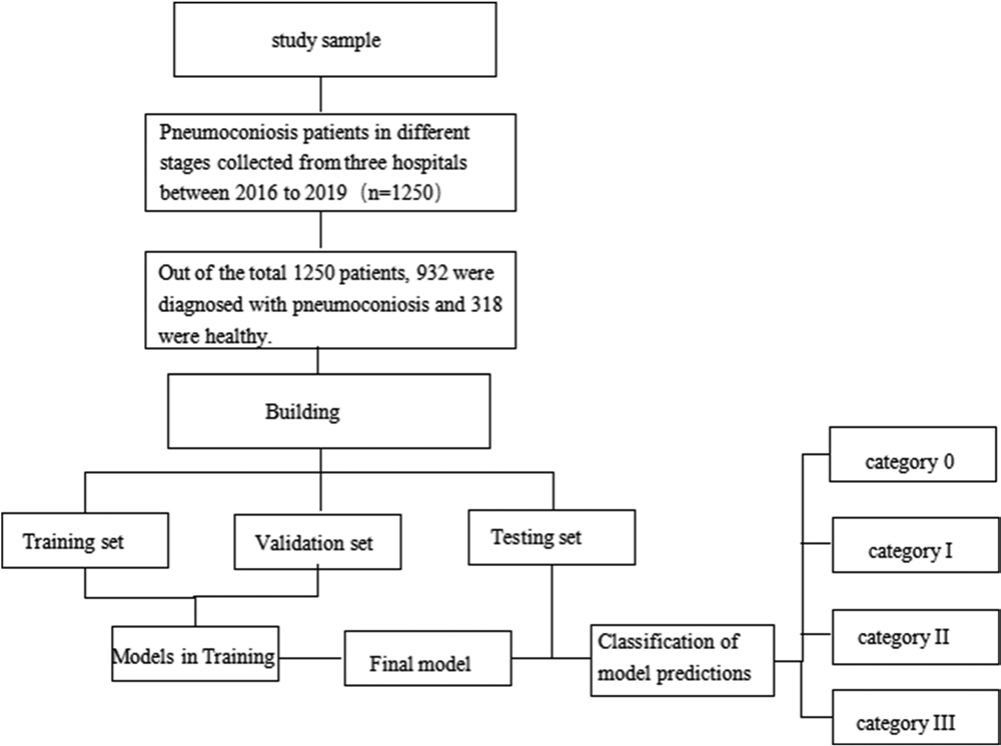
The outline of the study design.

In this study, we developed an efficient deep learning method that eliminates the need for manual feature recognition, reducing the workload of diagnosing pneumoconiosis (the outline of the study design is given in Fig. 1). This method can not only screen for pneumoconiosis but also stage its severity, providing a valuable guide for clinical diagnosis and treatment. What is more, we used the heat map as a highly effective method for quickly identifying abnormalities in computer-assisted diagnosis. The heat map is considered the most representative and helpful visualization tool for radiologists. Finally, we demonstrated the clinical usefulness of a deep learning model for classifying pneumoconiosis, following a comparative clinical analysis.

## 2. Materials and methods

### 2.1. Study population

All private information was de-identified. All participants were industrial workers with a history of DR screening of dust exposure for pneumoconiosis from 2016 to 2019. Of these participants, 932 were diagnosed with pneumoconiosis and 318 were healthy. The study comprised 124 women and 1126 men, including 23 women and 295 men in the healthy category. Their experience of dust service ranged from 1 to 40, with a mean duration of 20.56 ± 2.5 years. We did not exclude patients with emphysema, tuberculosis, bronchiectasis, or other structural lung diseases. This is because patients with pneumoconiosis may simultaneously experience these diseases.

### 2.2. Data acquisition

We retrospectively collected 1250 cases of different stages of pneumoconiosis from 3 hospitals. The patients’ details are summarized in Table 1.

**Table 1:** Summarizes demographic information of the patients.

Dataset origin	No. pneumoconiosis	No. normal	File type	Women	men	Category 0	Category I	Category II	Category III
Institution 1	569	209	DICOM	35	743	209	190	187	192
Institution 2	234	59	DICOM	56	237	59	92	70	72
Institution 3	129	50	DICOM	33	146	50	40	42	47

There were a total of 1250 cases (932 cases positive for pneumoconiosis and 318 healthy controls). “Positive cases” refer to cases that were positive for pneumoconiosis.

DICOM = Digital Communications in Medicine.

All images are presented in digital radiography.

Images were acquired using Siemens Axiom Aristors and Definium 6000 X-ray radiographers (General Motors). The digital radiography (DR) image settings were as follows: 120 kVp and automated mAs; 120 kVp and 250 mAs. The final digital images were generated using a DR workstation. Image processing techniques, such as edge enhancement and noise reduction, were turned off in the post-processing software. These images were calibrated to comply with the Digital Imaging and Communications in Medicine standard.

### 2.3. Annotation

Following the ILO guidelines, 3 board-certified radiologists reviewed the images and classified them into 4 categories as follows: 0 (n = 318), I (n = 322), II (n = 299), and III (n = 311); during the diagnosis, the profusion, shape, and size of pneumoconiosis lesions were considered the most important evidence for classifying pneumoconiosis.^[29,30]^ All radiologists were qualified for diagnosing pneumoconiosis, with >10 years of experience. In addition, 1 radiologist participated in the formulation of the latest diagnostic standard for pneumoconiosis in China, the “Diagnostic Standard for Occupational Pneumoconiosis GBZ70-2015.”^[31]^ We followed the strategy used by other researchers and divided the patients into 2 categories, namely normal and pneumoconiosis, by combining the I, II, and III categories into 1 category.^[32,33]^ The absence of disagreement on the classification of an image required multiple rounds of discussion and adjudication until complete agreement was reached.^[34]^ The categories of pneumoconiosis are provided in Appendix E1, Supplemental Digital Content, http://links.lww.com/MD/M857.

### 2.4. Deep learning models

Before training the CNNs, all images were processed, including down-sampling, histogram equalization, blank area removal, and down-sampling. Post-processing, the images were resized to a 256 × 256 pixel matrix, converted to a JPG format, and inputted to the server to build the dataset (Appendix E2, Supplemental Digital Content, http://links.lww.com/MD/M858). In this study, deep learning models were built using 3 CNN architectures as follows: ResNet50, ResNet101, and DenseNet. We use the original models without modification. The FLOPS(G) of these models were 3.8, 7.6, and 5.69, respectively, with ResNet101 having the highest FLOPS parameter.

### 2.5. CNNs training and validation

Before CNN training, we randomly split all data into a training set (80%) and test set (20%). Remaining 20% of the images from the training set were extracted as the validation set.

During the training, the parameters were continuously adjusted followed by adding the validation set. We adjusted the network structure and training parameters until the training accuracy of the model and the number of training sessions were maximized. In the experimental process, categorical-cross-entropy was used as the loss function, whereas rectified linear activation unit was used as the activation function to adjust the network parameters and fit the training data. Indicators, such as accuracy curves and loss rate curves, were monitored to observe changes in each parameter and to understand the training situation. Post training, we evaluated the effectiveness of the model by relevant evaluation indexes using the test set. The modeling strategy also mimicked the clinical diagnostic procedures, thus allowing us to merge pneumoconiosis-related knowledge into the artificial intelligence model. In addition, we requested 3 radiologists to independently read the images in the test dataset and compare the clinical diagnoses with that of the deep learning approach.

### 2.6. General scheme of the 4 pneumoconiosis classifications

**Figure 2.**
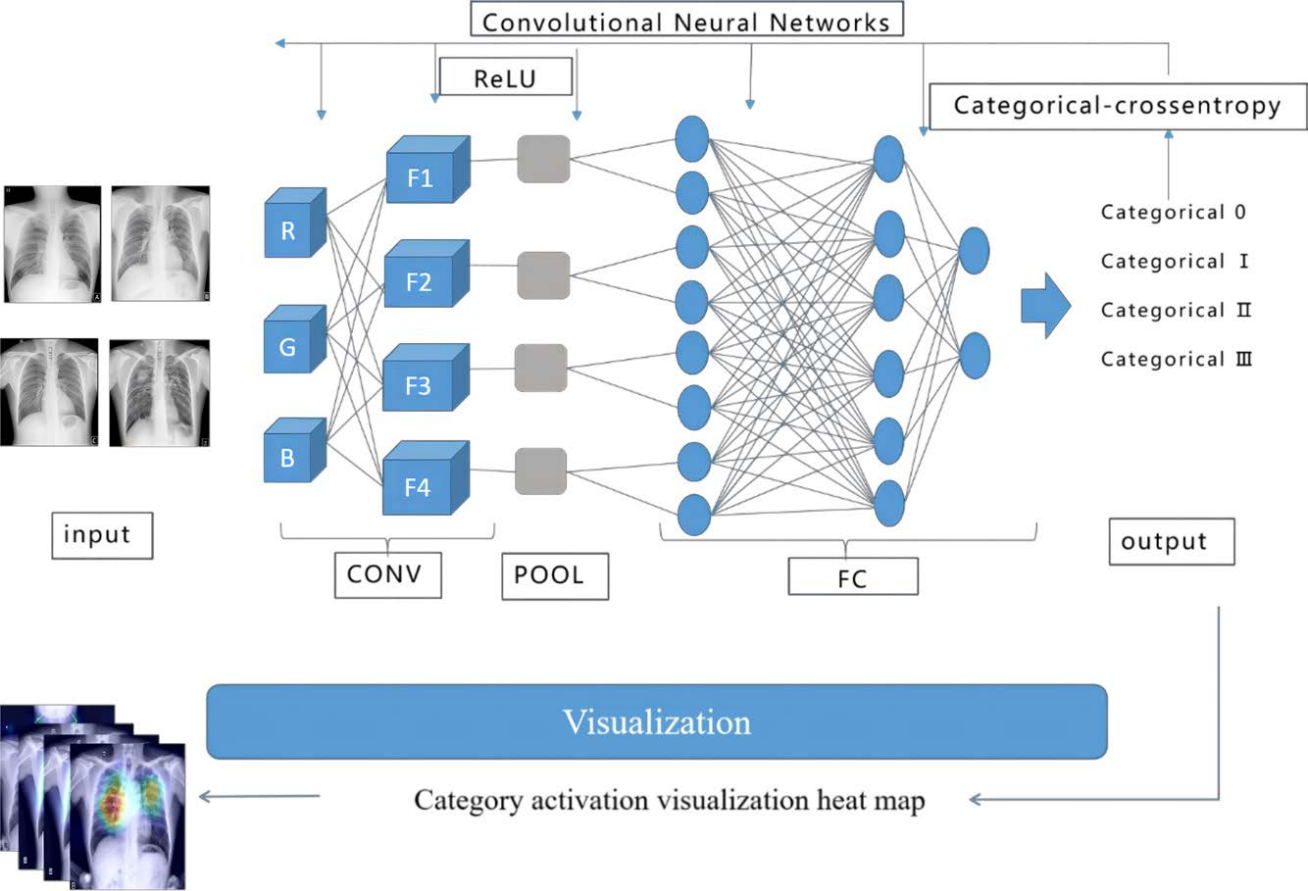
Schematic flow chart illustrating the procedure for the 4 classifications of pneumoconiosis.

Figure 2 depicts the general scheme of classification using a quadruple classification method. The network model was trained and supervised using the known labeled pneumoconiosis images. The backpropagation algorithm continuously adjusted the parameters of the network model to achieve the function of accurately classifying unknown images into 4 categories of DR images, that is, categories 0, I, II, and III.

### 2.7. Evaluation metrics

The accuracy, precision, and recall were used to evaluate the performance of the model. The evaluation indicators are presented in Appendix E3, Supplemental Digital Content, http://links.lww.com/MD/M859.

A cross-validation approach was used to evaluate the performance of the proposed deep learning model, and 4 expected results for true positive, true negative, false positive, and false negative were obtained.

A multi-categorization task necessitates the use of macro-P, macro-R, macro-F1, and macro-averages evaluation indexes. Data used in this study were divided into 4 categories. The 4-category problem was transformed into 4 binary problems, followed by the calculation of the check-all rate of the confusion matrices. The evaluation indicators are listed in Appendix E3, Supplemental Digital Content, http://links.lww.com/MD/M859.

### 2.8. Statistical analyses

We used the ROC analysis and AUC to measure the diagnostic effectiveness of the classifier. We conducted a nonparametric ROC^[35]^ analysis on an independent test dataset to evaluate the performance of the predictive models. Each point on the ROC curve represented a sensitivity/specificity pair corresponding to a specific decision threshold. In addition, we invited 3 radiologists (R1, R2, and R3) with >10 years of experience in pneumoconiosis diagnosis and familiarity with pneumoconiosis diagnostic criteria. These certified radiologists independently read the images in the test dataset in a double-blinded manner and classified them into categories 0, I, II, and III.

We evaluated the performance of the reader using the ROC analysis and compared it with that of the deep learning algorithm. Moreover, we conducted statistical analyses to evaluate the consistency between model classification and clinical evaluation using Kappa coefficients. We determined the model classification accuracy using IBM SPSS V.20 software.

## 3. Results

### 3.1. Experimental results

**Figure 3.**
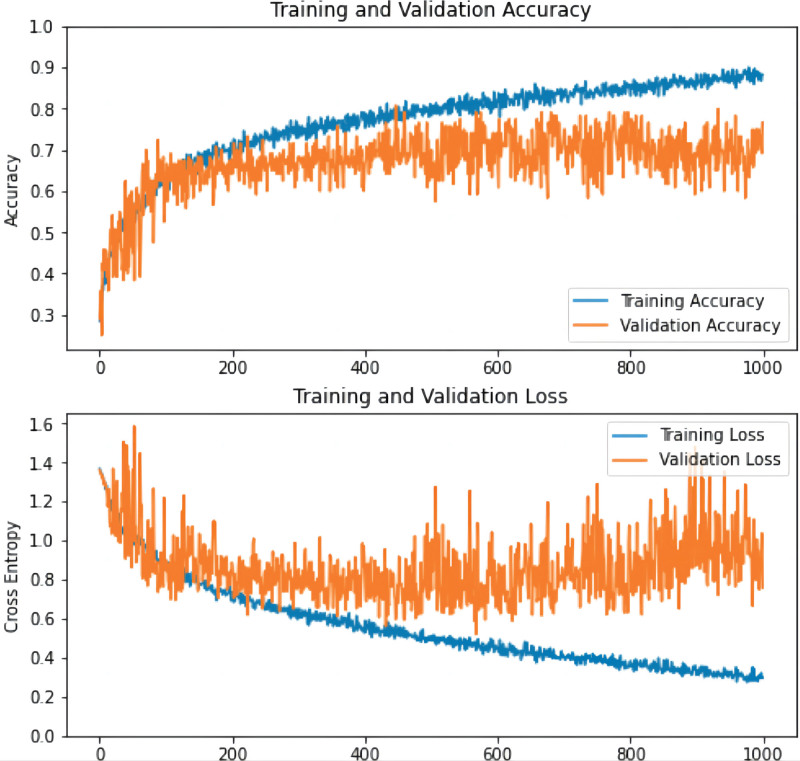
The accuracy and loss rate of the training process. The abscissa represents the smooth times, and the ordinate distinguishes the accuracy (see image above) and the loss rate (see image below), respectively, which increases over time, the accuracy on the training set and the verification set will increase, and the loss rate will decrease. As expected, there is a reduction of loss over the course of training as accuracy improves. The loss on the validation is similar to the training, which indicates that there is no appreciable overfitting. These training curves are used for model selection. In this case, the best performing model at epoch 1000 was used on the test data for final assessment. Val = validation.

As observed from the curves, the accuracy rate increased and the loss rate decreased in the validation and training sets until the network converged with the highest accuracy rate and the lowest loss rate (Fig. 3).

**Figure 4.**
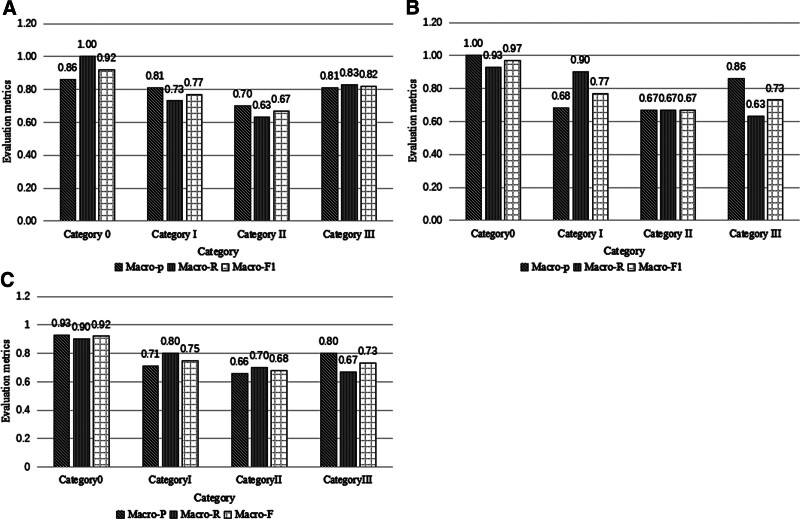
Evaluation metrics of 3 deep learning models for each category of pneumoconiosis. (A) ResNet50 convolution neural network. (B) Evaluation index of ResNet101 convolution neural network. (C) Evaluation index of DenseNet convolution neural network.

Figure 4 and Table 2 present the classification results of each dataset for patients with pneumoconiosis in ResNet50, ResNet101, and DenseNet CNNs.

**Table 2:** Overall indicators of the model.

	Macro-P	Macro-R	Macro-F	Accuracy
ResNet50	0.80	0.80	0.80	0.80
ResNet101	0.80	0.78	0.78	0.78
DenseNet	0.77	0.77	0.77	0.77

**Figure 5.**
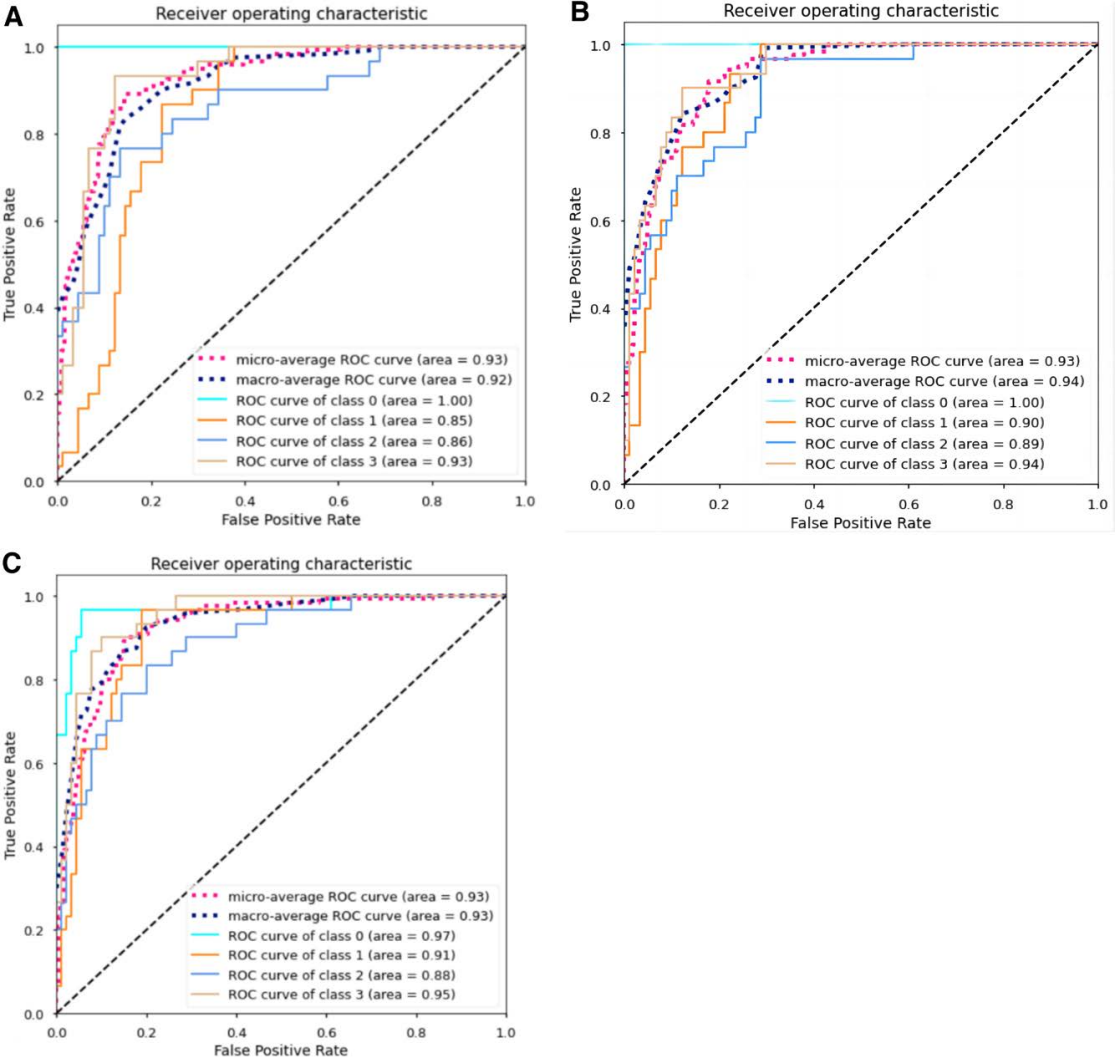
Receiver operating characteristic (ROC) curve of different CNNs. (A) ROC curve of ResNet50. (B) ROC curve of ResNet101. (C) ROC curve of DenseNet. CNNs = convolutional neural networks.

To evaluate the CNN performances, we plotted ROC curves. Figure 5A to C depicts the ROC curves of the 3 CNNs, which were subsequently summarized. The trained ResNet101 model demonstrated the best performance. The AUC values of Resnet101 micro-average and macro-average were 0.93 and 0.94, respectively, with AUC values of 1.0, 0.90, 0.89, and 0.94 for category 0, category I, category II, and category III, respectively.

### 3.2. Diagnostic performance compared with that of radiologists

The inconsistency rate between the model and the clinical assessment of pneumoconiosis classification was principally distributed in assessments for categories I/III and II/III, whereas the rate was lower for 0/I and other categories (0.016 and 0.033, respectively). Table 3 provide the distribution of inconsistent rates between ResNet101 and clinical assessment.

**Table 3:** The distribution of inconsistent rates between ResNet101 and clinical assessment.

	Model versus Clinical
Absolute inconsistency rate	0.72 (86/120)
0/I Inconsistency rate	0.016 (2/120)
I/II Inconsistency rate	0.083 (10/120)
II/III Inconsistency rate	0.083 (10/120)
Two-degree Inconsistency rate	0.033 (4/120)
Total	1 (120/120)

The model assessed pneumoconiosis classification with high accuracy and good agreement, with an overall accuracy and kappa values of 0.72, 0.98, 0.711, and 0.955 for the quadruple classification, dichotomous classification, quadruple classification Kappa value, and dichotomous classification Kappa value, respectively. Table 4 provide the accuracy and consistency of ResNet101 evaluation.

**Table 4:** The accuracy and consistency of ResNet101 evaluation.

	Classification accuracy (quadruple classification)	Classification accuracy (dichotomous classification)	Kappa value (quadruple classification)	Kappa value (dichotomous classification)
Model versus Clinical	0.72	0.98	0.711	0.955

### 3.3. Visual heat map analysis

**Figure 6.**
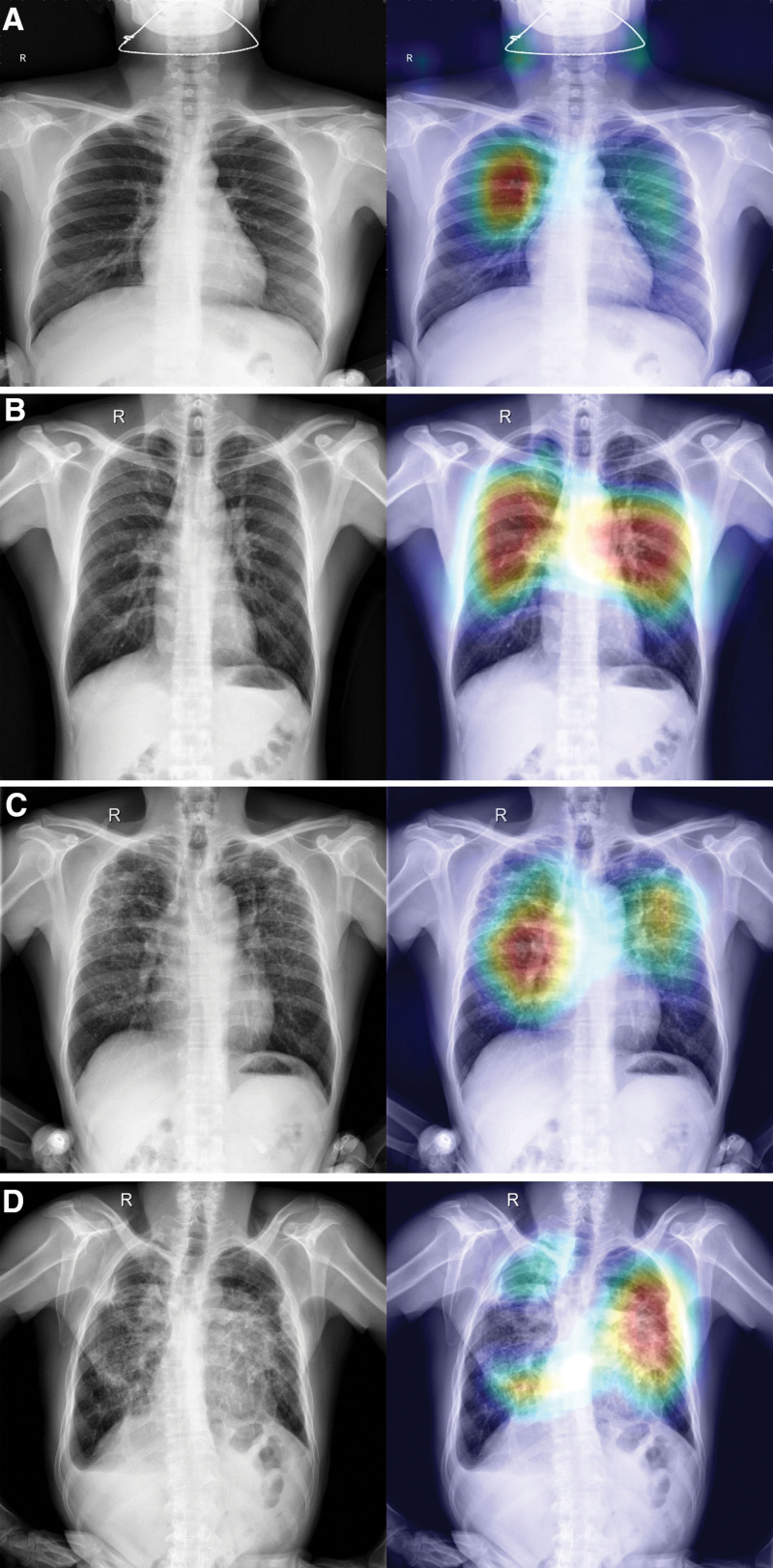
Different stages of pneumoconiosis and their corresponding category activation visualizations. (A) X-ray images and the corresponding heat maps: (a) pneumoconiosis stage 0 of X-ray image, (b) heat map of (a). (B) X-ray images and the corresponding heat maps: (a) pneumoconiosis stage I of X-ray image, (b) heat map of (a). (C) X-ray images and the corresponding heat maps: (a) pneumoconiosis stage II of X-ray image, (b) heat map of (a). (D) X-ray images and the corresponding heat maps: (a) pneumoconiosis stage III of X-ray image, (b) heat map of (a).

The aforementioned network could present visualization results, and generate the visualization result map of the ResNet101 model classification output, that is, the category activation visualization heat map. Different stages of pneumoconiosis and their corresponding category activation visualizations are presented in Figure 6A to D. We evaluated the learning ability of the model using a heat map and the overlay presentation of original images. For relevant features of pneumoconiosis images, brighter the color, higher the possible value of the predicted pneumoconiosis lesion. The red area represented the closest value to which the network predicted relevant features of the pneumoconiosis image. This corresponds to the staging of pneumoconiosis given by our radiologists.

## 4. Discussion

According to ILO standards, pneumoconiosis diagnosis and staging is determined by the degree of filling in small opacities, the presence of large opacities, and the aggregation of small opacities on Dr. This conventional diagnostic process is subjective and time-consuming, leading to misclassification and unreliable results. The problem mentioned above becomes more severe and occurs more frequently during pneumoconiosis screening programs in underdeveloped areas. We developed a deep learning approach that automatically evaluates pneumoconiosis on chest Dr. Our approach provides screening results and identifies the stages of pneumoconiosis. Additionally, it offers a detailed visual interpretation of the predictions, which helps increase confidence in the classifier’s ability to solve black box problems.^[36]^

Our proposed deep learning-based pneumoconiosis screening model differs from other models in that it directly extracts features from the input image, reducing the workload of feature extraction and human intervention. Additionally, we not only screened participants for pneumoconiosis but also performed 4 classifications to guide diagnosis and treatment. Furthermore, this study improved the reliability of pneumoconiosis classification by offering a visual interpretation of the model, thus increasing transparency. Our findings may also serve as a roadmap for unlocking the “black box” principle of artificial intelligence for image analysis tasks in other medical fields. Eventually, through a comparative analysis of the model and clinical assessment, this study further confirmed the clinical utility of the model in pneumoconiosis classification.

We employed 3 convolutional neural models to screen and stage pneumoconiosis, which was categorized into 4 levels (0–III). Among the models, ResNet101 proved to be the most suitable for pneumoconiosis screening and classification. It achieved AUC values of 1.0, 0.90, 0.89, and 0.94 for categories 0, 1, 2, and 3 respectively. ResNet101 also outperformed models used in previous studies. Okumura et al^[37]^ developed a CAD system based on the rule of ILO and an artificial neural network (ANN) power spectrum analysis of pneumoconiosis. The results of classifying normal pneumoconiosis with abnormal pneumoconiosis demonstrated mean AUCs of 0.93 and 0.72 on chest X-ray films in the highest category (severe pneumoconiosis) and lowest category (early pneumoconiosis), respectively. Subsequently, Okumura et al^[38]^ developed a CAD system based on ANN classification of the textural features of pneumoconiosis chest films. The image database consisted of 36 chest films divided into 4 categories, ranging from 0 to 3. The AUC values for category 3 pneumoconiosis and category 0 pneumoconiosis were 0.89 ± 0.09 and 0.84 ± 0.12, respectively. We compared the accuracy and consistency of deep learning models with those of the relative gold standard, where that the model classification accuracy was 0.80, compared with the clinical standard classification. The kappa value and accuracy of the quadruple classification were 0.733. The accuracy and kappa value of the 2 classifications were 0.98 and 0.931, respectively. Wang et al^[39]^ explored the potential of deep learning in assessing pneumoconiosis, as revealed by digital chest radiographs, and compared their performance with radiologists. They used the inception-V3 Network. The model’s AUC was 0.878, whereas that of the 2 radiologists were 0.668 and 0.772, respectively. Moreover, the readers displayed moderate agreement (κ = 0.423, *P* < .001). However, Wang et al only screened patients for pneumoconiosis. Only few researchers have performed a quadruple classification of pneumoconiosis,^[40]^ where our proposed model performed better.

The deep learning-based model proposed in this study has few differences compared to other models for pneumoconiosis screening. First, we utilized the heat map method of result visualization, which is considered the most representative and helpful method for readers to identify abnormalities through computer-assisted diagnosis quickly. This method also provides intuitive information about the confidence level of the test. Furthermore, it has been demonstrated that using heat maps significantly improves the detection rates of lesions found in individual abnormalities.^[41]^ Second, our study eliminates the need for manual annotation, reducing the workload of feature extraction and manual intervention. Our deep learning model achieves a higher screening accuracy compared to other studies. Our model achieves an accuracy of 0.98, which is higher than previous studies: 0.92,^[16]^ 0.94 to 0.95,^[32]^ and 0.97.^[40]^ Additionally, Yang et al^[42]^ study also reported an accuracy of 0.92. Although our results are only slightly higher than Zhang study, we have a larger sample size with more stage II and III cases, making our findings more accurate and comprehensive. Table 5 constructs a comparative table between the current study and other state-of-the art studies. The evaluation indexes of our model are slightly different from other scholars” studies, probably due to the difference in sample size, the dataset of our study is the largest, so the results are more precise, and the evaluation indexes of our study are broader and more comprehensive.

**Table 5:** Comparative study with state-of-the-art studies.

Study	Model	Purpose	Dataset size	Accuracy	AUC (%)	Macro-P	Macro-R	Macro-F	References
Devnath et al^[43]^	CheXNet	Screening	71	90.2	–	–	–	–	43
	DenseNet			87.58	–	–	–	–	
	InceptionV3			87.58	–	–	–	–	
	Xception			86.27	–	–	–	–	
	Resnet50			83.66	–	–	–	–	
Hao et al^[30]^	ResNet34	Screening	142	89.3	–	–	–	–	30
	DenseNet53			88.6	–	–	–	–	
Sun et al^[44]^	AED-Net	Classifying	527	90.4	–	–	–	–	44
Wang et al^[39]^	Inception-V3		923	–	–	–	–	–	39
Yang et al^[42]^	ResNet		1237	92.46	–	–	–	–	42
Zhang et al^[40]^	ResNet	Classifying	512	97.3	–	–	–	–	40
		Screening		92.7	–	–	–	–	
Devnath et al^[45]^	CheXNet		71	92.68	–	–	–	–	45
Current study	Resnet50	Classifying and screening	1250	80	0:100	80	80	80	
					I:90				
					II:89				
					III:94				
	Resnet101			78	0:97	80	78	78	
					I:91				
					II:88				
					III:95				
	DenseNet			77	0:100	77	77	77	
					I:85				
					II:86				
					III:93				

Our study had some limitations. First, the dataset only included chest X-ray images. Despite chest radiography being the standard method for pneumoconiosis diagnosis, X-ray chest films have other characteristics, such as insufficient resolution, overlap effect, and factors with skeletal (noise) images. The next step involved designing a network structure and data preprocessing process that was suitable for the aforementioned characteristics. CT usually provides details of shadowed areas within the lungs. Some countries have introduced regulations for high-resolution CT as a diagnostic criterion for pneumoconiosis. Considering this future trend, shadowed areas should be tested in future studies as patients with pneumoconiosis cannot undergo pathology tests and open-chest examinations. The criteria used in this study were relatively subjective gold standards. We selected patients with pneumoconiosis and healthy individuals for model training. The diagnosis of patients with pneumoconiosis should be combined with relevant dust reception history and laboratory tests to provide a definite diagnosis. However, it is not possible to distinguish pneumoconiosis from other lung diseases with similar imaging signs. This will be the next research direction to open new horizons.

## 5. Conclusions

In conclusion, this study proposed a deep learning-based model for detecting and classifying pneumoconiosis cases using DR images. We proposed a fully automated end-to-end architecture model that did not require manual feature extraction. It could perform binary and multi-classification tasks with accuracies of 98% and 72%, respectively. This model could further simulate the diagnostic behavior of the radiologists. The system can be used in remote areas of pneumoconiosis-affected countries to overcome the shortage of radiologists. In addition, these models can be used to diagnose other diseases associated with the chest, including tuberculosis and pneumonia. Therefore, it is worthwhile to develop further deep learning solutions for pneumoconiosis screening and classification in clinical practice.

## Author contributions

**Conceptualization:** Yajuan Zhang, Bowen Zheng, Weiguo Chen, Long Li.

**Data curation:** Yajuan Zhang, Bowen Zheng, Fengxia Zeng.

**Funding acquisition:** Yajuan Zhang, Long Li.

**Investigation:** Yajuan Zhang, Bowen Zheng, Tianqiong Wu.

**Methodology:** Yajuan Zhang, Bowen Zheng, Yuli Peng.

**Writing – original draft:** Yajuan Zhang.

**Writing – review & editing:** Yajuan Zhang, Bowen Zheng, Long Li.

**Formal analysis:** Xiaoke Cheng, Jiefang Wu.

**Project administration:** Yonliang Zhang.

**Resources:** Yuanlin Xie, Wei Yi, Long Li.

**Supervision:** Long Li.

## Supplementary Material






