# Supplementary material for: Potential of digital chest radiography-based deep learning in screening and diagnosing pneumoconiosis: An observational study
Source: Medicine (Baltimore). 2024 Jun 21;103(25):e38478. doi: 10.1097/MD.0000000000038478 (PMC11191863; doi:10.1097/MD.0000000000038478)
Supplement: Supplementary file 1 [file medi-103-e38478-s001.docx]

# Appendix E1

The pneumoconiosis imaging reports and Diagnostic Standard for Occupational Pneumoconiosis GBZ70-2015 standards are as follows:

To maintain consistency in diagnosing pneumoconiosis readings, the films were read by three highly qualified physicians according to the diagnostic criteria of Occupational Pneumoconiosis GBZ70-2015. The results of their agreement were considered the relative gold standard.

The rules of the final category according to GBZ70-2015 were determined as follows:

| Category 0 | No turbidity found or grade 1 turbidity in one division |
| --- | --- |
|  |  |
| Category I | Grade 1 massive turbidity in more than two subregions or  Grade 2 massive turbidity in four or fewer subregions |
|  |  |
| Category II | Grade 2 or more turbidity in four or more subregions or  substantial turbidity in Grade 3 |
|  |  |
| Category III | Large opacities presented with a long diameter  of not less than 20cm and a short diameter of more than 10cm |
