# Supplementary material for: Potential of digital chest radiography-based deep learning in screening and diagnosing pneumoconiosis: An observational study
Source: Medicine (Baltimore). 2024 Jun 21;103(25):e38478. doi: 10.1097/MD.0000000000038478 (PMC11191863; doi:10.1097/MD.0000000000038478)
Supplement: Supplementary file 2 [file medi-103-e38478-s002.docx]

# Appendix E2

The input images for this study were images. Considering diverse shooting environments, different images often have varied contrast, brightness, and other attributes. Ensuring the consistency of the model input images and the effectiveness of the model operation necessitates pre-processing the pneumoconiosis camera images to reduce the images with irrelevant factors on the classification results. The pre-processed pneumoconiosis Digital Imaging dataset possesses good classification properties.

Acquire

image

Store

classification

Down-sampling,

Training models

Histogram equalization

Blank area removal

**Date preprocessing flowchart**

Image store: Pneumoconiosis DR images that met the inclusion criteria were collected and uniformly stored in a Digital Imaging and Communications in Medicine format.

Image classification: Different stages of pneumoconiosis images are stored in varied file types.

Down-sampling: The images were resized to a 256×256 pixel matrix. This significantly reduced the burden on the hardware, minimized the amount of computation, and facilitated the effective training of realistic models.

Histogram equalization: To facilitate image feature presentation, we enhanced the images such that the local gray contrast increased. Histogram equalization is a common method of image enhancement.

Blank area removal: We eliminated irrelevant information and retained useful information.

The pre-processed pneumoconiosis DR images increased the contrast of the internal structure of the lung field and displayed good classification characteristics.

The development platform and hardware platform for this study were as follows:

We used Python 3.6, CUDA 9.0, JupyterLab, SimplelTK, Scipy, Numpy, OpenCv, TensorFlow, Keras, and Matplotlib as the major tool systems used for image processing, model building, statistical analysis, and plotting.

The flat key platform for this study was an HP-Z420 server equipped with 1080Ti graphics, CPU: Intel(R) Xeon(R) CPU E5-1650 0 @ 3.20GHz; RAM: 64G; and GPU: GeForce GTX1080Ti.
