# Supplementary material for: Potential of digital chest radiography-based deep learning in screening and diagnosing pneumoconiosis: An observational study
Source: Medicine (Baltimore). 2024 Jun 21;103(25):e38478. doi: 10.1097/MD.0000000000038478 (PMC11191863; doi:10.1097/MD.0000000000038478)
Supplement: Supplementary file 3 [file medi-103-e38478-s003.docx]

# Appendix E3

In this experiment, we assessed the prediction ability of the model using the evaluation indexes of accuracy, precision, and recall.

Macro-R and Macro-F1 were defined as：

**Precision (P):**

$\text{P}=\frac{\text{TP}}{\text{TP}+\text{FP}}$ (1)

**Recall (R):**

$R=\frac{TP}{TP+FN}$ (2)

**F1-Score:**

$F1=\frac{2PR}{P+R}$ (3)

**Accuracy:** $Accuracy=\frac{TP+TN}{TP+FP+TN+FN}$ (4)

The classification effectiveness of a model not only should be judged by its classification of a category but also by its inclusion of all four categories. Macro-averaging and micro-averaging evaluate the classification effectiveness of a model from a global perspective, including the metrics of all four categories.

Macro-P, Macro-R, and Macro-F1 were defined as follows:

$Macro-P=\frac{P_{0}+P_{I}+p_{II}+P_{III}}{4}$ (5)

$Macro-R=\frac{R_{0}+R_{I}+R_{II}+R_{III}}{4}$ (6)

$Macro-F1=\frac{{F1}_{0}+{F1}_{I}+{F1}_{11}+{F1}_{III}}{4}$ (9)

Macro-averages represent the arithmetic means of all categories, including *macro-P*, *Macro-R*, *R*, and *Macro-F1*.
